# A 2D virtual reality system for visual goal-driven navigation in zebrafish larvae

**DOI:** 10.1038/srep34015

**Published:** 2016-09-23

**Authors:** Adrien Jouary, Mathieu Haudrechy, Raphaël Candelier, German Sumbre

**Affiliations:** 1 École Normale Supérieure, PSL Research University, CNRS, Inserm, Institut de Biologie de l’ENS (IBENS), F-75005 Paris, France; 2 Sorbonne Universités, UPMC Univ. Paris 06, UMR 8237, Laboratoire Jean Perrin, F-75005 Paris, France

## Abstract

Animals continuously rely on sensory feedback to adjust motor commands. In order to study the role of visual feedback in goal-driven navigation, we developed a 2D visual virtual reality system for zebrafish larvae. The visual feedback can be set to be similar to what the animal experiences in natural conditions. Alternatively, modification of the visual feedback can be used to study how the brain adapts to perturbations. For this purpose, we first generated a library of free-swimming behaviors from which we learned the relationship between the trajectory of the larva and the shape of its tail. Then, we used this technique to infer the intended displacements of head-fixed larvae, and updated the visual environment accordingly. Under these conditions, larvae were capable of aligning and swimming in the direction of a whole-field moving stimulus and produced the fine changes in orientation and position required to capture virtual prey. We demonstrate the sensitivity of larvae to visual feedback by updating the visual world in real-time or only at the end of the discrete swimming episodes. This visual feedback perturbation caused impaired performance of prey-capture behavior, suggesting that larvae rely on continuous visual feedback during swimming.

Early behavioral theories described complex goal-driven behaviors as reactive strategies, where behavior is generated only in response to an ever-changing external environment^1^. Therefore, behavior could be decomposed into a sequence of stimulus-response associations, where the neuronal mechanisms underlying these sensorimotor transformations could be studied in open-loop conditions. However, current theories have demonstrated that this is not the case^2^, even in simple invertebrate animals^3^,^4^. Instead, in order to reach a goal, animals rely on the feedback resulting from their actions. Unlike open-loop conditions where external cues are independent from the animal’s actions, in behaving animals, actions shape the perception of the environment. Motor-command signals are combined with sensory signals caused by the action of the body on the environment. Together, these signals allow for a continuous evaluation of the relevance of the motor commands.

Virtual reality (VR) systems provide the opportunity to investigate the role of sensory feedback during goal-driven behaviors, by allowing the experimentalist to artificially perturb or alter the sensory feedback. In closed-loop VR, the environment is continuously updated according to the animal’s motor responses. Since in VR, the animal’s head is restrained, VR systems are compatible with high-resolution functional neuronal recordings, and suitable for studying the neuronal basis of goal-driven behaviors. VR has been successfully applied to study two-dimensional place cells in the rat^5^, path integration in the fly^6^, and motor adaptation in the zebrafish^7^.

Zebrafish is an emerging animal model for studying the neuronal circuit mechanisms underlying goal-directed behaviors. At 6 days post fertilization (dpf), the larva already displays a rich repertoire of visually guided motor behaviors such as prey tracking^8^, the optomotor response^9^ and phototaxis^10^. In walking animals, leg movements can be measured using a spherical treadmill in head-fixed preparations^11^. However, inferring the displacement intended by the tail movements of a zebrafish larva is not a straightforward task. In the past, two options have been used to record motor activity in zebrafish. The first option relied on “fictive swim”, which involves recording, in paralyzed larvae, the activity of a bundle of motor neuron axons^7^,^12^ as an indication of locomotion. Larvae turn using asymmetric tail oscillations. Thus, the direction of the intended movement was obtained by comparing the intensity of the signal recorded from both sides of the tail. Using this method, larvae can perform phototaxis and the optomotor response in a virtual 2D environment^13^. Alternatively, it is possible to monitor tail movements using a high-speed camera when the larva’s head is restrained in low-melting agarose^14^. There are several advantages to inferring the larva’s motion directly from the tail movement kinematics, rather than using “fictive swim”. First, due to the variation in the positioning of the electrodes, the fictive swim readout must be calibrated regularly. Second, recording tail movements allows classification of tail movements according to the animal’s natural behavioral repertoire^15^, or for fine tracking of tail kinematics^16^. Third, neuromuscular-blocking drugs can affect the normal activity of neuronal circuits (unpublished data). Finally, in addition to visual feedback, tail movements provide proprioceptive feedback, absent in paralyzed larvae. Previously, a method for VR using tail-movement kinematics to adjust the visual feedback was developed for the study of unidirectional displacement triggered by whole-field motion stimuli. The difference between successive images of the tail produced 1D forward navigation in the virtual environment^14^. Another method was adapted to provide predefined feedback independent of the kinematics of the tail at the onset of the tail movement^17^. In the latter, the visual feedback was not linked to the kinematics of the tail movements but only to its onset time.

In contrast to these approaches, here we propose a method capable of providing two-dimensional feedback in real time, adapted to different types of tail movements. In order to relate the tail kinematics to the intended displacements of the larvae, we generated a library of larvae movements in free-swimming conditions, containing a diverse sample of the larva’s behavioral repertoire. This library enabled us to extract the relationship between tail kinematics and the resulting change in orientation and position of the larva. In head-restrained larvae, this readout was computed in real time to readjust accordingly the visual environment displayed around the larva. Our VR approach enabled the larva to interact meaningfully with its environment in different behavioral contexts. The larvae could change their swimming direction to follow a whole-field motion, and track small moving virtual prey. Perturbation of the delay of the visual feedback affected the success rate of capturing the virtual prey, suggesting that larvae rely on a continuous feedback for navigation. The behavioral database and the code required to infer the trajectory from the tail movements were written in a Python Jupyter Notebook^18^. The code controlling the visual feedback was written in C++. They are all open source and can be downloaded and modified as required (www.zebrain.biologie.ens.fr/codes)

## Results

### Prediction of the larva’s trajectory from the kinematics of tail movements

Zebrafish larvae navigate by producing discrete stereotypical tail movements called swim bouts. In agarose-restrained larvae, the typical frequency of tail oscillations during a bout, is 20 to 30 Hz^19^. In order to provide real-time feedback, tail kinematics should be filmed at high-acquisition rates (typically above 200 Hz). Therefore, the processing of the acquired images must be computed in just a few milliseconds. The Reynold’s number of swimming larvae ranges between 50 and 900 Re, which puts them in a transitional flow regime^20^, thus neither inertial nor viscous forces can be neglected. This situation is unlike adult fish that swim in flow regime, where approximations could enable the real-time computation of the thrust generated by the tail movements^21^. Real-time computation of the thrust in transitional flow regime is, so far, technically impossible.

**Figure 1:**
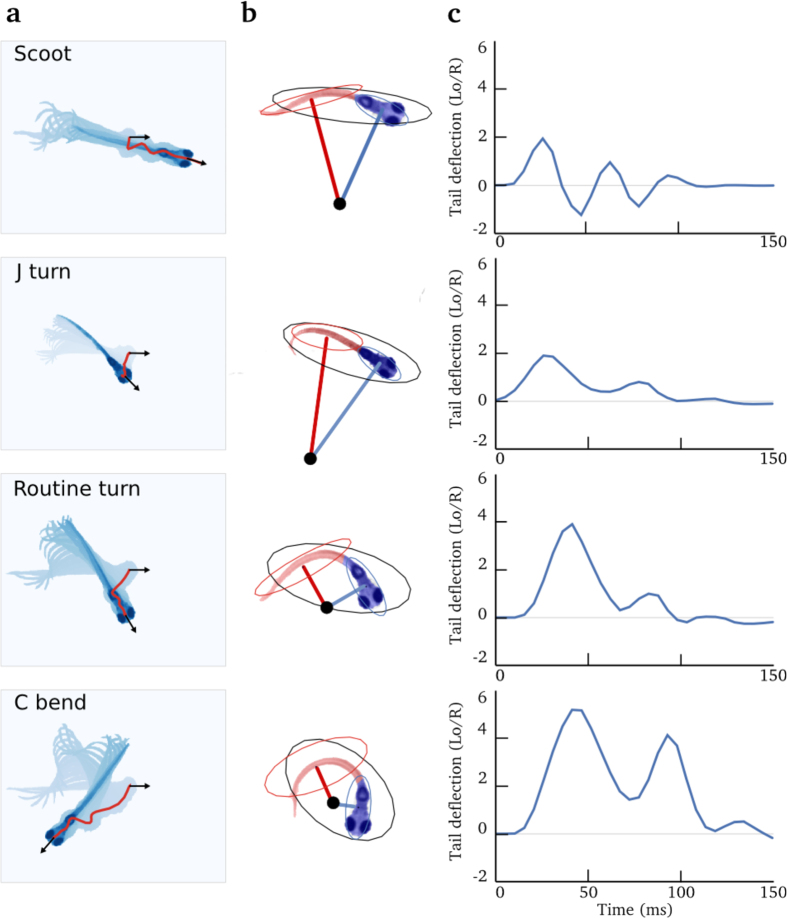
Quantification of tail movements in free-swimming conditions. Row depict movements from different categories. **(a)** Superimposition of the image of a larva during a tail bout. The first image is in light blue and successive images are darker. The path followed by the head is shown by a red line, the black arrows represent the head orientation at the beginning and end of the bout. **(b)** Illustration of the image processing method to a characteristic snapshot of the movement in **(a)**, an ellipse was fitted on the binarized image of the larva (in black). Pixels were split in two groups according to the major axis of the black ellipse: pixels shown in red or blue, superimposed on the larva. For each of these two groups of pixels, a second ellipse was fitted (red and blue ellipse) and the corresponding minor axes were drawn in red and blue. The center of curvature (black dot) was defined as the intersection between the two minor axes. The deflection was defined as the inverse of the average distance between all the pixels in the larva and the center of the curvature (1/*R*). To obtain a dimensionless value, the result was multiplied by the length of the larva at rest *L*_*o*_. The sign of the deflection was computed as left = negative and right = positive. **(c)** The resulting deflection of the tail over time, for each of the different types of movements in **(a)**.

**Figure 2:**
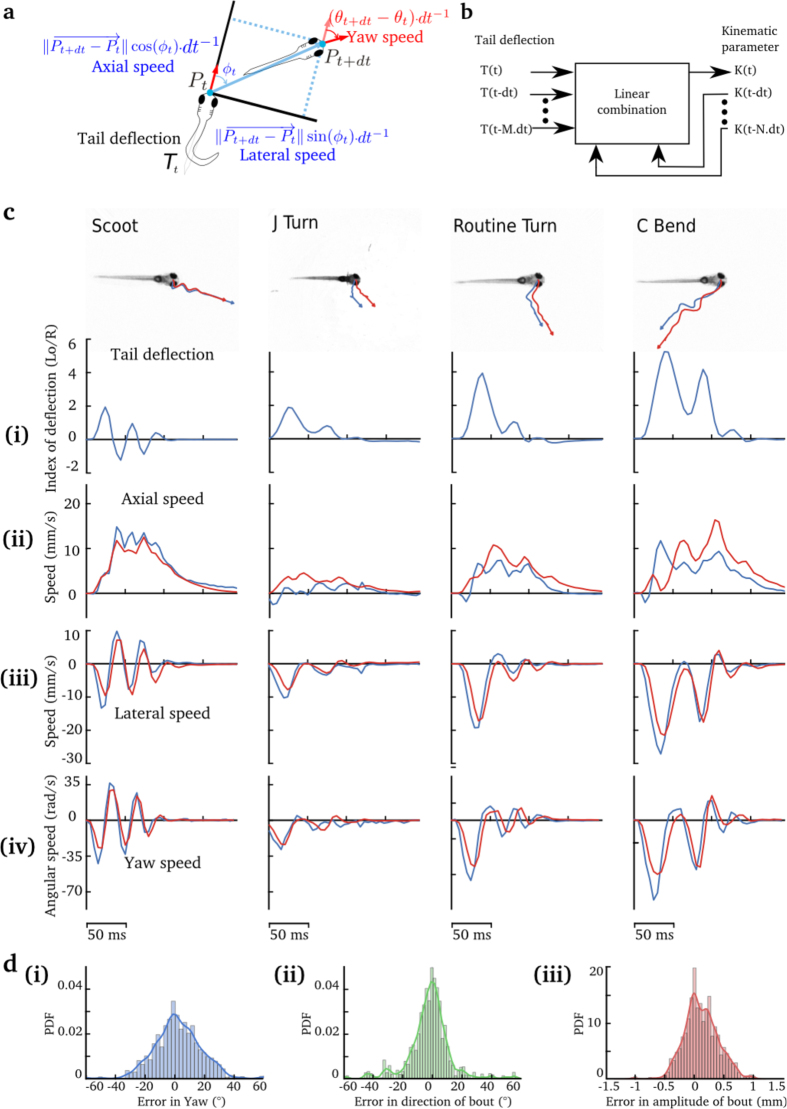
Prediction of the larva’s trajectory from the deflection of the tail. **(a)** Parametrization of the displacement of the larva in the horizontal plane. Only 3 parameters are required to describe the trajectory: the axial, lateral and yaw speed. **(b)** Illustration of the Auto-regressive Model with External Input. Each of the three parameters of trajectory was computed using the tail deflection. Each of the kinematic parameters at time t, *K*(*t*) was computed using a linear combination of its past values: 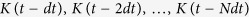 and the present and past values of the tail deflection: 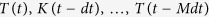. See Materials and Methods for details. **(c)** Examples of four different types of movements showing the true path of the larva (in blue) and that predicted (in red). **(i)** Tail deflection corresponding to different categories of movement. **(ii)** The axial speed. **(iii)** The lateral speed. **(iv)** Yaw angle. For each case, the observed kinematic parameter is in red, and the predicted in blue. **(d)** Distribution of the errors between the predicted position and orientation, and those observed, for: **(i)** change in head orientation; **(ii)** direction of movement; **(iii)** amplitude of the tail bout. The results presented in **(c)** and **(d)** were taken from the test dataset.

To predict trajectories from tail kinematics, we used a data-driven approach to learn the relationship between the tail movement kinematics and the fish displacement in the horizontal plane (two dimensions). We recorded the displacement and tail kinematics from free-swimming larvae to generate a library of tail movements (see Materials and Methods). Our library of movements consisted of ~300 tail bouts from 6–8 dpf nacre larvae. The shape of the tail was quantified by computing the tail deflection^22^ (see Fig. 1b and Materials and Methods). Figure 1 shows the time series of the tail deflection associated with stereotypical movements. This quantification of tail kinematics was fast (we used a C++ written algorithm capable of analyzing the tail movements at 200 Hz), and it resulted in a low-noise, smooth oscillating time series. To describe the change in orientation and position of the larvae in the swimming plane, we used three parameters: axial, lateral and yaw speeds (Fig. 2a). Figure 2c shows the kinematic parameters of free-swimming larvae associated with four previously described maneuvers: scoot, J turn, routine turn and C bend^23^. These maneuvers have distinct kinematics and correspond to different behavioral contexts (e.g. J turn is associated with hunting and C bend with escape from predators). Kinematic parameters were chosen to be smooth oscillating times series during swim bouts.

In order to establish the relationship between the oscillating tail deflections and changes in orientation and position of the larva, we used an auto-regressive model with external input (ARX Model). This technique can predict the value of a kinematic parameter (axial, lateral and yaw speed) using a linear combination of both its past value and the past and current values of the tail deflection (see Fig. 2b and Materials and Methods). Thus, a simple regression is needed to fit the relationship between the tail deflection and the resulting trajectories. To assess the significance of our model, we predicted the trajectories in the test dataset of free-swimming larvae (a random set of 20% of the tail bouts in our library), based only on the changes in tail deflection. The resulting trajectories were then compared to the actual trajectories of the larvae. Figure 2c shows that the trajectories resulting from different categories of tail movements can be fitted using the same model. Due to error accumulation, the trajectory predicted from the tail deflection may sometimes diverge from the observed trajectory, but the overall kinematics were similar. The quality of the prediction of the final orientation and position after a tail bout is shown in Fig. 2d. To compute the error between the predicted and the observed paths, we used a bootstrap between the test and the training datasets, in order to get a reliable estimate of our error. The mean square error (MSE) in the prediction of the direction of movements was 19.4° (Fig. 2d.ii), a similar MSE of 23.4° was observed in the prediction of the change in the direction of the larva’s head (Fig. 2d.i). The MSE in the prediction of the larva’s displacement was 0.3 mm representing 1/10 of the body length of the larva (Fig. 2d.iii). Moreover, we showed that our method was able to generalize by predicting trajectories of tail movements absent in the training set (see Materials and Methods), thus confirming that the ARX method captures the most relevant dynamics of the larva’s locomotion.

To create the visual VR system, larvae were head-restrained in a drop of low-melting agarose and placed in a recording chamber (see Materials and Methods). The tail movements were then filmed with a high-speed camera at a frame rate of 200 Hz. Larvae do not track moving gratings faster than 10 Hz^24^. Thus, a video projector with a refresh rate of 60 Hz was adequate for the visual temporal acuity of the zebrafish larva. We computed the time lag of the feedback loop using the following procedure. We modified our C++ VR routine to provide a simple feedback: if the camera sensed higher light level (when a LED was turned on), the monitor screen switched from black to white. We then used two photodiodes and an oscilloscope to compute the delay between the onset of the LED and the onset of the screen illumination. Using this approach, we estimated a latency of 70 ± 10 ms (mean ± s.t.d).

Using the ARX model, we inferred the changes in kinematic parameters resulting from tail movements in real time and updated accordingly the patterned visual stimuli projected around, or below the larva’s recording chamber. The VR software was based on a custom program written in C++, using OpenCV to process images and OpenGL to display the visual environment. The routine required for providing the visual feedback in real time is available at (www.zebrain.biologie.ens.fr/codes). Due to the flexibility of this method, we were able to study different types of visual behaviors. All routines required for the analysis of the library of movements and the generation of the ARX model were programmed in a Python Jupyter Notebook. This approach enables others to reproduce the data analysis, and to easily adapt the code according to their own needs. As a proof of principle, we tested the VR system using two different goal-directed visual behaviors: the optomotor response and prey-capture behavior.

### Optomotor response in a two-dimensional visual virtual reality system

When presented with a whole-field coherently moving visual stimulus, zebrafish larvae turn and swim in the direction of the perceived motion. This behavior, known as the optomotor response (OMR), allows the larvae to maintain a stable image of the world on the retina, and thus, a stable position with respect to their visual environment. For example, OMR could prevent larvae from being carried downstream by water currents. The OMR can be reliably evoked in the larvae from 5 dpf and it is maintained throughout the entire lifespan of the fish^25^.

Based on previous studies, we chose a grating velocity of 1 cm/s and a spatial frequency of 1 cm projected on a screen placed 0.5 cm underneath the larva^13^,^14^. At the beginning of each trial, the angle between the initial orientation of the grating and the head direction of the larva was randomly chosen between −180° and 180°. During the stimulation, the speed and orientation of the grating were updated in real time according to the larva’s tail kinematics (see Supplementary Movie 1). Each experiment consisted of 120 trials, where each trial was composed of periods of visual stimulation (6 s) and periods in which the grating was steady (20 s). For the data analysis, we only considered and analyzed trials where larvae generated at least one tail bout. The analyzed trials represented 40 ± 7.5% (mean ± s.t.d) of all trials.

**Figure 3:**
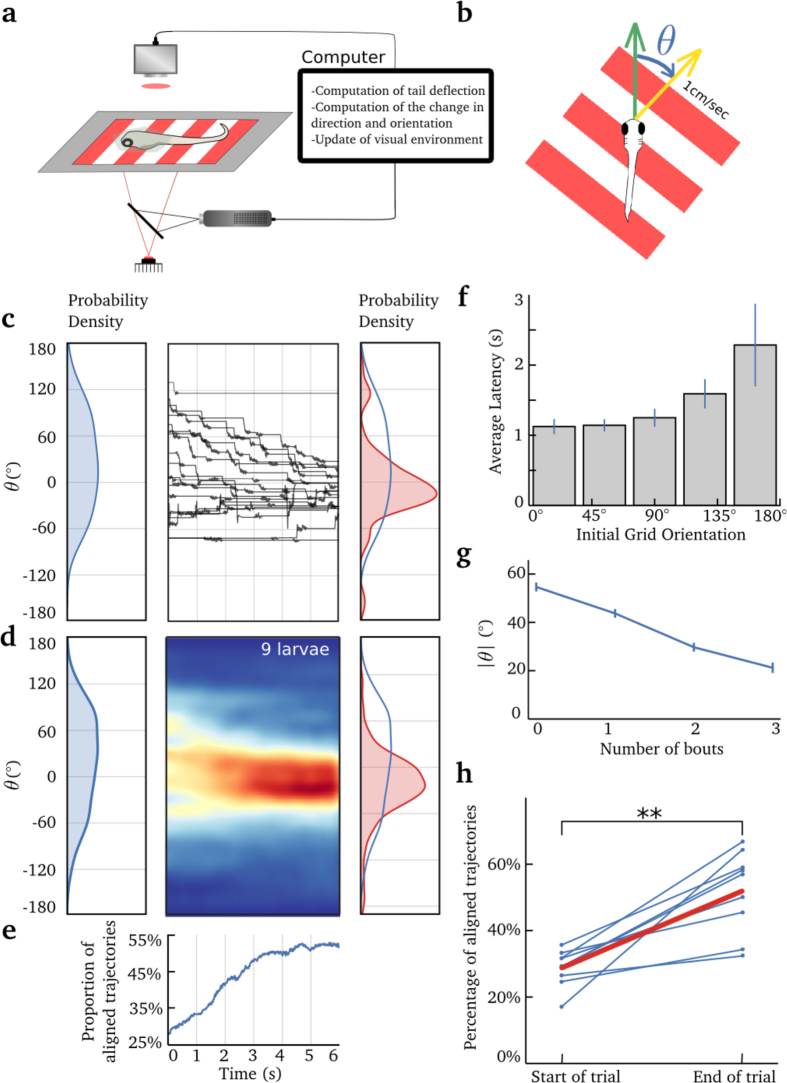
The optomotor response in virtual reality. (**a**) A schematic of the experimental setup. The tail was imaged using a high-speed camera, an IR LED for illumination, and a high-pass filter to prevent the visual stimulus from reaching the camera. A projector was used to display the moving grating on a diffusive screen placed 0.5 cm below the larva. The larva was head-embedded in low-melting agarose at the bottom of a petri-dish. The tail was free to move. **(b)** The grating moved at 1 cm/s. *θ* represents the difference between the larva’s heading direction (green arrow) and the direction of the moving grating (yellow arrow). **(c)** Center panel: example of the changes in *θ* for one larva (20 trials). Left panel: Initial distribution of *θ* for the same larva. Right panel: Final orientation (*θ*_*t*=6s_) distribution. The initial orientation distribution (*θ*_*t*=0_) is superimposed in blue for comparison. **(d)** Left panel: Initial orientation distribution for all larvae and all trials for which at least one bout was generated. Center panel: color-coded density of trajectory as a function of time for the 6 s trials. Left panel: Initial distribution of *θ*_*t*=0_ for all larvae. Right panel: Final orientation distribution (*θ*_*t*=6*s*_) for all larvae. **(e)** Proportion of larvae aligned with the moving stimuli (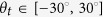) as a function of time during the trial. The time scale is common to **(c)**, **(d)** and **(e)**. **(f)** Histogram of latency as a function of the initial orientation of the grating, error bar indicates s.e.m. **(g)** Average of |*θ*|, for successive bouts, error bar indicates s.e.m. **(h)** Average percentage of trajectories aligned with the moving stimulus (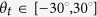), at the beginning and at the end of the trials, for each larva. The average is shown in red.

When larvae were initially aligned with the direction of the moving gratings, they displayed shorter latencies (Fig. 3f) and swam at an average speed of 0.43 ± 0.25 cm/s in the direction of the moving stimulus (mean ± s.t.d, N = 148 trials, from 5 larvae, Supp. Fig. 2c). The larva’s ability to follow and align itself with the whole-field moving stimulus was improved by applying a gain of 3 to the axial speed. This improvement could be explained by the change in tail dynamics when the head is restrained in agarose^19^, and/or the lack of feedback from other sensory modalities (e.g. the lateral line). Larvae produced on average 3.26 ± 1.8 bouts per trial (mean ± s.t.d, N = 546 trials, from 9 larvae) and the average bout duration was 313 ± 8 ms (mean ± s.e.m, N = 1783 bouts, from 9 larvae), which is consistent with previous reports^19^. The distribution of angles between the larva and the stimulus direction decreased with time (Fig. 3c,d). Successive bouts brought the angle of the larva’s head to an average deviation of 20° ± 2.1° (mean ± s.e.m) with respect to the axis of displacement of the moving grating (Fig. 3g). We considered that a larva was aligned with the direction of the stimulus motion if the angle of its head and the grating motion was lower than 30° (chosen to approximately match the average deviation at the end of the trials, 36.4°, all trials considered). The proportion of aligned larvae increased by two-fold during the 6 s trials (from 28.2% to 51.6%, N = 546 trials, from 9 larvae, Fig. 3e,h).

Because this alignment could result from feedforward motor commands that do not necessarily rely on feedback, we performed a control experiment to test the effect of the visual feedback on the larva’s behavior. At the beginning of each trial, the direction of the larva’s head was aligned with the movement of the grating. Each experiment was composed of 50 trials in open-loop conditions interleaved with 50 trials in closed loop where the speed and orientation of the grating were updated in real time according to the tail kinematics of the larva. Each trial was composed of periods of visual stimulation (10 s) and periods in which the grating was steady (20 s). In open-loop conditions, the speed of the larvae in the virtual environment increased at the beginning of the trials and decreased after a few seconds (Suppl. Fig. 2a,d). Moreover, the number of scoot tail movements associated with forward swim was significantly higher in the closed-loop conditions (see Materials and Methods and Suppl. Fig. 2e). Because forward scoots have longer durations than turns, the average bout duration was longer when larvae were initially aligned with the direction of the whole-field moving stimulus (517 ms) than in the conditions where the larvae were initially set a random angle with respect to the direction of the moving stimulus (330 ms). The bout durations were shorter in closed-loop (517 ms) than in open-loop conditions (770 ms, Suppl. Fig. 2b, N = 5, p = 0,04, Wilcoxon signed-rank test). This difference in bout duration was already significant at the first bout (536 ± 189 ms in closed loop and 1296 ± 572 ms in open loop, N = 5, p = 0.03, Wilcoxon signed-rank test). Additionally, we observed that larvae reacted to the absence of feedback by increasing the intensity of their tail oscillations during the first bout (Supp. Fig. 2g–f). In certain cases, the lack of feedback led to an abrupt change in tail kinematics during the first bout (Supp. Fig. 2f). These results confirm previous findings^14^, and further suggest that within the framework of the OMR, larvae can integrate visual feedback during a bout and react to it by modulating tail deflections adequately (e.g. increase in duration of the bout, interruption or even modulation of amplitude and orientation of the tail oscillations during the first bout, Supp. Fig. 2).

Overall, these results confirm that the trajectory reconstructed from tail movements can be used to provide visual feedback in real time, enabling larvae to orient according to moving visual stimuli, in a 2D visual virtual environment.

### Prey capture in a two-dimensional visual virtual reality system

At 5 dpf, zebrafish larvae start hunting prey. This behavior is critical for survival and relies on several decision-making processes. The first step is visual recognition. Larvae rely mostly on vision to capture prey, as demonstrated by the dramatic decrease in the number of prey eaten in the dark^16^,^26^. Prey capture can also be induced in free-swimming or head-restrained larvae when presenting small moving dots (~4° in size) in the larva’s field of view^15^,^27^,^28^,^29^,^30^. This size has been shown to optimally elicit prey capture^15^,^27^,^28^ and match the natural neuronal circuit tuning of the larva’s optic tectum^15^,^28^,^30^, the highest visual processing area necessary for visually guided prey detection and capture^26^. As observed for free-swimming larvae, prey capture in head-restrained conditions showed similar locomotor and oculomotor movements intended to bring the larva in front of the virtual prey^17^,^27^. In contrast, large dots elicited turns away from the stimulus^27^,^29^,^31^,^32^,^33^. After detection, the larva initiates a series of bouts to precisely and progressively orient towards and approach the prey. During prey capture, the larva adapts the speed, intensity and directionality of its movements based on the updated position of the prey^16^.

**Figure 4:**
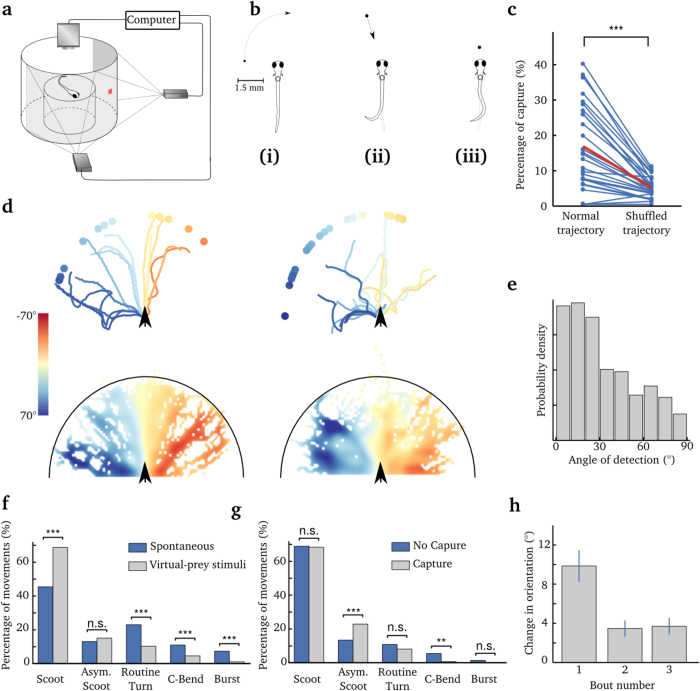
Prey-capture behavior in virtual reality. **(a)** Schematic of the experimental setup. The larva is positioned on an elevated stage in the center of a cylindrical recording chamber. Visual stimuli were projected on a screen surrounding the recording chamber, covering a field of view of 180°, and centered around the direction of the larva’s head. The tail was imaged using a high-speed camera mounted on a binocular. For illumination, an IR LED was placed below the chamber. Two projectors were used to project the virtual prey, each covering a field of view of 90°. **(b)** Presentation of the virtual environment during each trial. (i) The virtual prey of 4° appeared from either side of the larva with an angular speed of 20°/s. (ii) After the onset of the first tail bout, the angular speed of the virtual prey was set to 0°/s and its position on the screen was further updated according to the larva’s tail movements only. (iii) A trial was considered successful if the larva got at least 400 *μ*m from the virtual prey. **(c)** Percentage of trials that ended in successful capture of the virtual prey. Only trials where larvae executed at least one tail bout were considered. Left: Each dot represents the performance of individual larvae. Right: The performance obtained by shuffling the angular positions of the virtual prey in each dataset. The red segment depicts the average. (*p* = 1.4*10^−5^, Wilcoxon signed-rank test). **(d)** Examples of paths of a larva towards the virtual prey. The paths are color-coded according to the position of the virtual prey at the onset of the first tail bout (color bar). Upper panel: Individual paths for one larva. Left: Paths leading to capture. Right: paths failing to capture the virtual prey. Lower panel: Superposition of the trajectories from all larvae (N = 27). Each bin of the meshgrid is color-coded according to the average position of the virtual prey for the trajectory in that bin (color bar). Left: paths leading to capture. Right: Paths failing to capture the virtual preys. In all panels, the black arrows indicate the initial position of larvae. **(e)** Distribution of the angle of the virtual prey at the bout’s onset in the first trial. **(f)** Proportion of bouts in each category of movement, during the trials (virtual-prey stimuli) and between trials (spontaneous). Not Significant: *p > 0.05, *p* < 0.05, **p < 0.01, ***p < 0,001. **(g)** Proportion of bouts in each category of movement for successful or unsuccessful trials. **(h)** Change in head orientation for the first three bouts. Only trials in which larvae performed at least three bouts were considered. Error bar: s.e.m.

Under head-restrained conditions, we reproduced the orientation and pursuit maneuvers toward the virtual prey, in a visual virtual environment (Fig. 4a,b). No increase in gain was applied for this experiment because prey capture relies on small-amplitude movements that are less affected by embedding the head of the larvae in agarose^19^. Experiments consisted of 166 trials. Each trial mimicked a situation where a 100 *μ*m light spot appeared 1.5 mm away from the larva. In this configuration, the apparent angle of the virtual prey (4°) optimally elicited a prey-capture behavior^15^,^27^. The larva was head-restrained in a drop of low-melting agarose and placed in an elevated stage in the center of a cylindrical recording chamber. At the beginning of each trial, we projected on the circular screen a 4° circular black spot moving on a white background at an angular speed of 20°/s along the azimuthal plane. The black spot began first appeared at ±90° relative to the longitudinal axis of the larva, and moved towards 0° (rostrally).

Immediately after the onset of the larva’s first tail bout, the angular speed of the prey in the virtual environment was set to 0°/s. Any further changes in size and position of the black circle projected on the screen depended on the predicted trajectory of the larva. Figure 4b and Supplementary Movie 2 illustrate the experimental design. If the larva oriented itself toward the virtual prey, the black spot was then projected towards the center of the larva’s field of view in accordance with the predicted change in yaw, and its radius was increased as the larva swam in its direction. We considered that a larva captured the virtual prey if its trajectory in the virtual environment reached at least 400 *μ*m from the virtual prey (beyond this point, larvae usually execute a maneuver to swallow the prey based on ram or suction^16^). A trial ended after a successful capture, or when the angle between the larva’s head and the virtual paramecium exceeded ±90°, the latter representing a failure.

We found that larvae produced at least one tail movement in 14% of the trials (13.8%, N = 6750 trials, from 27 larvae), which is consistent with previous reports^17^,^27^,^30^. Figure 4f shows that the tail movements produced during the trials were based more on forward scoots and less on large amplitude movements (routine turns, C bends and bursts) compared to the movements spontaneously produced between trials. This is consistent with the fine maneuvers required to catch the virtual prey.

In trials where at least one tail movement was generated, larvae were able to capture virtual prey on average 16.1% of the time (166 trials per larva, 27 larvae), and up to 40% for the best performing larvae (Fig. 4c). Successful captures of virtual prey were associated with an increase in asymmetric scoot movements (Materials and Methods and Fig. 4g), a category of movements including small amplitude turns such as J turns. As a control experiment, we shuffled the trajectories of the larvae in VR with respect to the position of the virtual prey. For each larva, control paths were generated by associating paths in VR with random virtual-prey locations within the larva’s field of view (±90°). Then, we computed the average percentage of captures for the shuffled trajectories. Random trajectories could reach the target in only 4.8% of the cases, compared to 16.1% (Fig. 4c, *p* = 1.4*10^−5^, Wilcoxon signed-rank test).

Larvae preferentially initiated the first tail bout when the prey was at a ±30° angle in their field of view (Fig. 4e). The larvae performed an average of 3.6 ± 2.6 bouts (mean ± s.t.d, N = 99 successful trials, from 27 larvae) to capture the virtual prey, compared to 4.4 bouts in free-swimming conditions^17^. For cases in which larvae failed to capture the virtual prey, their paths were still oriented toward the final target (Fig. 4d). As observed in freely swimming larvae^16^, the first bout coarsely brought the paramecia in front of the larva, and successive finer correcting bouts (including J turns) brought the paramecia progressively closer (Fig. 4g,h). The duration of bouts was 191 ± 29 ms (mean ± s.t.d, N = 27 larvae) as previously reported (188 ms^16^), which is significantly shorter than those observed during OMR (313 ms).

These experiments show that the prey-capture behavior in the 2D visual VR can reproduce previously described characteristics of prey-capture behavior in freely-swimming larvae, thus validating the use of this visual VR system to study the role of visual feedback during fine goal-driven behavior.

### Integration of visual information during tail bouts

The visual system could use two potential strategies to provide information about the executed movements. The relative position of external landmarks before and after a bout can provide visual feedback on the result of a motor action. An alternative strategy is to have a continuous update on the action rather than a discrete one, by integrating the angular speed of the visual environment during the movement itself. Computing the cumulative rotation (before and after the movement) would, however, require the visual system to integrate over a large angular displacement and extremely high velocities (the amplitude of oscillations of the head during a turn, can reach velocities of up to 4000°/*s*, Fig. 2c.iv). Previous studies have reported that the larva uses visual feedback following the end of the bouts to compare the observed and the expected position^17^. In this previous study, a predefined feedback was presented at the onset of a tail movement independently of its kinematics. In contrast, our method provides a continuous update of the larva’s trajectory according to the tail kinematics.

**Figure 5:**
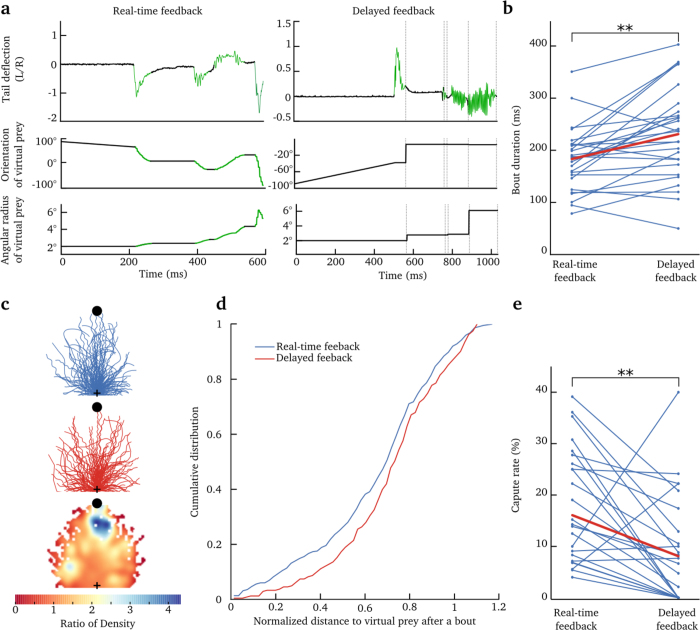
Delayed visual feedback affects prey-capture behavior. **(a)** Tail deflection of the larva and the corresponding modification of the virtual-prey position (visual feedback): angular position and size of the virtual prey. Left panel: feedback was continuously updated during tail movements (green). Right panel: the feedback was delayed and presented only after the end of the bout (the time indicated by the vertical dashed lines). The green segments on all curves indicate the detection of tail bouts and the corresponding modification in size and position of the virtual prey. **(b)** Average duration of bouts in real-time and delayed feedback conditions, for each larva. The average is shown in red. (*p* = 0.0012, Wilcoxon signed-rank test). **(c)** Normalized bouts: the paths of each bout was rotated and rescaled according to the position of the virtual prey (black dot). The starting position is indicated by the black cross. Upper panel: normalized bouts for all larvae when the feedback was presented in real time. Middle panel: normalized bouts of all larvae, when the feedback was presented at the end of a bout (delayed feedback). Lower panel: ratio of the density of normalized bouts. The color bar indicates the ratio of density between the real-time and delayed feedback conditions (>1 indicates that the density of paths is larger for the real-time with respect to the delayed feedback) **(d)** Cumulative distribution of the normalized distance to the virtual prey at the end each bout, for trials in which the feedback was provided in real time (blue) or after the end of the bout (red). The distribution were significantly different (*p* = 0.04, Kolmogorov-Smirnov test). A normalized distance of 0.5 means that the bouts reduced the distance to the prey by half. **(e)** Percentage of trials that ended in a successful capture of the virtual prey, for real-time feedback (left), and delayed feedback trials (right) (from 27 larvae). The red segment depicts the average.

To test whether visual feedback is used by the larva during the generation of bouts, we altered the visual feedback provided during the swim bouts. More specifically, we performed experiments in which the feedback was updated only at the end of the bout, when the speed was slower than 0.2 mm/s (Fig. 5a and Supplementary Movie 3). This perturbation was introduced in one-third of the trials (83 trials per larva, 27 larvae). In comparison to trials in which visual feedback was provided in real time, the visual-feedback-delayed trials resulted in longer bout durations: 215 ± 42 ms vs. 191 ± 29 ms (mean ± s.t.d, *p* = 0.0012, Wilcoxon signed-rank test, N = 27 larvae; Fig. 5b). Although we did not find significant differences in the categories of movement produced in the two conditions, individual bouts were less successful at reaching the virtual prey in the delayed feedback condition (Fig. 5c,d, the normalized distance to the virtual prey after a bout is 0.66 ± 0.27 in real-time feedback conditions and 0.73 ± 0.24 in delayed-feedback conditions (mean ± s.t.d), p = 0.04, Kolmogorov-Smirnov test). This subtle change in the visual feedback also decreased the percentage of capture by half from an average of 16% to 8% (Fig. 5e, p = 0.002, Wilcoxon signed-rank test).

Moreover, we did not observe significant differences in the inter-bout-interval (IBI) between the real-time (1.23 s ± 1.10 s (s.t.d)) and delayed-feedback conditions (1.30 s ± 1.08 s (s.t.d)). In contrast to previous studies^17^, the paths generated by the larvae were unique and highly variable. This could explain the observed large variability in the IBI and account for our inability to detect significant changes in the IBI between the two conditions.

Overall, these findings suggest that the zebrafish larvae are capable of integrating visual information during movements and not only in-between bouts as previously suggested^17^.

## Discussion

In contrast to studies in natural conditions, VR systems enable the manipulation of visual feedback, and therefore are ideal to study its role during goal-directed behaviors. To generate the visual VR system, we first determined the speed and orientation of free-swimming larvae from their tail kinematics. For this purpose, we used an autoregressive model fitted on a relatively small library (approximately 300 tail bouts) in order to relate a tail movement to the trajectory of the larvae. As a proof of principle, we first tested our VR system using a whole-field moving stimulus (grating) capable of inducing OMR. Under these conditions, larvae were able to align and swim in the direction of motion using tail bouts of relatively long durations (~300 ms). Larvae presenting initial deviations with respect to the direction of the grating’s motion were capable of aligning with the stimulus after 3 tail bouts. When they were aligned with the motion of the grating, the presence of visual feedback increased the proportion of forward scoot movements over turn movements. In open-loop conditions, larvae generated significantly longer bouts of increasing amplitude or could transition from scoot to burst regime within a single bout, as if they were searching for visual feedback. The same VR approach allowed us to study prey-capture behavior in a virtual environment. After detection of the virtual prey, larvae produced a fine tail movement that first coarsely aligned them with the moving virtual prey. Then, using on average two other short duration bouts (~190 ms), larvae were capable of reaching the virtual prey with a success rate of 16% and up to 40% for the best larvae. The paths were associated with fine reorientation maneuvers.

Our method reproduced features of free-swimming navigation during goal-driven behavior (e.g. duration of bouts and reduction in angle with respect to the virtual prey in the first bout). However, we found a difference in the frequency of emergence of spontaneous behavior. In head-restrained conditions, larvae generated fewer tail bouts than in free-swimming conditions: one bout every 1.22 ± 0.16 s in free-swimming conditions (mean ± s.t.d, from T. W. Dunn *et al.*^34^), in contrast with one bout every 43.3 ± 26.4 s (mean ± s.t.d, from previous study^35^) in head-restrained conditions. The difference was conserved despite the introduction of a visual-feedback loop in head-restrained conditions. This difference could emerge from the absence of sensory feedback from non-visual modalities (e.g vestibular, lateral line), or from the stress generated by the immobilization of the larvae.

In addition, the control of the visual environment allowed studying of the role of visual feedback on the behavioral performance of the larvae. Previous studies suggested that visual feedback is mostly used by the larvae at the end of the bout^17^. Here, we show that when the visual feedback was updated only at the end of a tail bout rather than in real time, individual bouts were longer and less precise in reaching the virtual prey with a 50% decrease in the prey-capture success rate. These results suggest that larvae are capable of integrating visual feedback and reacting within a swimming bout, despite their short durations and the larva’s large head-angular velocities. The discrepancy between our results and those of Trivedi *et al.*^17^ could emerge from a difference in the type of feedback provided during movement in the two studies. In the previous study, the paramecia disappeared during movement and reappeared at the end of the bout, at a predefined position. In our case, the virtual prey was always visible, thus providing more robust visual feedback. Overall, our experiments suggest that despite the short duration of the tail bouts, they can be modulated in real time according to the received visual feedback, rather than being feed-forward ballistic movements.

The zebrafish larva is a unique vertebrate model that enables the combination of optogenetics and single-plane illumination microscopy (SPIM) to monitor virtually whole-brain dynamics with near- or single-cell resolution^13^,^36^,^37^. To image the larva using SPIM, the larva needs to be head-restrained in low-melting agarose. Although tail movements can be monitored simultaneously, no visual feedback about its own acts is provided. The combination of SPIM and visual VR systems will enable monitoring whole-brain dynamics and behavior in more natural conditions, such as those encountered by the larva when freely swimming. In the future, this method could be used to study the neural mechanisms underlying fine goal-directed behaviors and error correction. Finally, the observation of motor actions in goal-driven navigation is limited by the level of locomotor activity in head-fixed larvae. Prey-capture behaviors could be triggered in only 14% of the trials, similar to previous reports^17^,^27^,^30^. In the future, these limitations could be improved by using multi-sensory stimulation^38^,^39^, or by combining the response of larvae with appropriate learning paradigms^40^. A similar methodology could also be applied in juvenile or adult zebrafish in order to study more complex cognitive processes such as social behaviors^41^,^42^ or place conditioning^43^ in VR conditions.

## Materials and Methods

### Zebrafish preparation

Experiments were performed on 6–8 dpf nacre larvae, a mutant lacking melanophores^44^. Embryos were collected, and raised at 28 °C in E3 embryo medium. Larvae were kept on a 14/10 h on/off cycle and fed with paramecia after 5 dpf. For VR experiments, larvae were embedded in low-melting agarose (2%) dorsal side up in the center of the circular recording chamber. After the agarose jellified, the chamber was filled with embryo medium. For OMR experiments, the agarose around the tail was removed up to the swim bladder, using a scalpel. For prey-capture experiments, the eyes were also free to move. All experiments were carried out in accordance with approved guidelines and approved by *Le Comité d’Éthique pour l’Éxpérimentation Animal Charles Darwin (03839.03).*

### Imaging of zebrafish movements

An IR LED (850 nm, IR dragon optic, Osram) was used to illuminate the larvae from below. For imaging the eyes and tail movements, we used a high-speed camera (200 Hz, M3 MotionScope, Redlake) mounted on a microscope (PZMIII-BS, World Precision Instrument). The setup was placed on an anti-vibration table (Kinetic System vibraplane 2212). In free-swimming conditions, the position and orientation of the larvae were computed by detecting the high contrast eyes of the nacre larva. The tail movements were quantified using the method presented in Fig. 1. An ellipse was fitted on the binarized image of the fish. Then the pixels of the larva’s image were split into two groups, according to the major axis of the ellipse, and an ellipse was fitted on each of the two sets of pixels. A center of curvature was defined as the intersection of the minor axis of the two ellipses. From the center, the deflection was defined as the inverse of the average distance between all the pixels in the fish and the center of curvature (1/*R*). The result was multiplied by the length of the fish at rest (*L*_0_) in order to obtain a dimensionless value. The Python code required to extract the paths and tail deflection from the video of a larva is available in an Jupyter Notebook (https://github.com/ajouary/VR_Zebrafish/blob/master/Code/FishTracking.ipynb) for reproducibility and reuse.

### Classification of tail bouts

This method uses fuzzy K-nearest neighbor to classify movements according to their similarity with respect to manually labeled tail bouts. We used the Dynamic Time Warping algorithm to compare the time series of tail deflections^35^. Movements were classified in five categories: scoot, asymmetric scoot, routine turn, C bend and burst. Asymmetric scoots included small amplitude turns such as J turns, O bends were grouped along with bursts. This method has a classification accuracy of 82%.

We pooled the movements generated by all larvae and used a Chi-squared test to compute the significance of the difference in the proportions of movements performed in each category for the different conditions (Fig. 4c,d and Supp. Fig. 2.e). We then applied a Bonferroni correction to adjust for multiple comparisons.

### Library of tail bouts in free-swimming conditions

We recorded the library containing in total ~300 tail bouts in 5 sessions. The videos were recorded at 200 Hz, with an exposure of 1 ms (M3 MotionScope, Redlake), in a field of view corresponding to 4 cm^2^ in the center of a petri-dish containing approximately 10 larvae. Prey-capture trajectories were recorded by introducing paramecia in one of the sessions. C bends and bursts occurred spontaneously, for instance when the wave generated by one larva triggered a startle response in a larva nearby. Only movements that started and finished inside the field of view were kept for further analysis. Although we did not identify individual larvae, we estimated that each larva would not contribute to more than ~5 bouts in the database. Supplementary Figure 1 shows the tail deflections of all the tail bouts in the library, classified according to the movement category they belong to.

### Visual stimulation

A pico-projector (refresh rate of 60 Hz, P4x, aaxa) was used for the visual stimulation projected on the diffusive screen (*N*° 216, White diffusion, Rosco Cinegel). For the OMR experiments in VR, the larva was immobilized the at the center of a petri dish. The stimulus consisted of a square wave grating with a spatial period of 1 cm, at the maximal contrast, projected on a screen placed 5 mm below the larva. For prey-capture experiments, the larva was positioned on an elevated stage within a cylindrical chamber of 5 cm diameters. The cylinder was surrounded by a diffusive screen. Two projectors were placed at ±45° relative to the larva’s head direction to create the visual environment. The position of the projectors was chosen to minimize the deformations generated by the curvature of the circular chamber.

### Autoregressive Model with External Input

The Autoregressive Model with External Input is a time-domain system identification model which has the following structure:


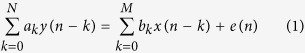


In our case, *y* represents the output kinematic parameters (axial, lateral or yaw speed), *x* is the input tail deflection and *e* is the error. *k* is an index variable corresponding to successive video frames acquired at 200 Hz. *a*_0_ = 1 ensures that the resulting system is causal (if *a*_0_ = 0, the past value of *y* depends on the current value of *x*). M and N represent the size of the memory for the input and output, respectively. The vector of unknown parameters we seek to identify is:


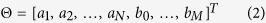


To identify the unknown variables, we started by observing the system at rest (before the onset of a tail bout). An input signal *x*(*n*) is then fed into the system, and the output *y*(*n*) is observed for the interval 0≤*n*≤*K*, where n is a temporal index and K is the total number of time steps in the time series:


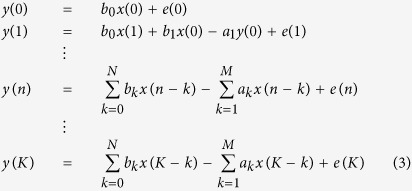


All these equations can be written as a large matrix equation:


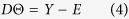


With:


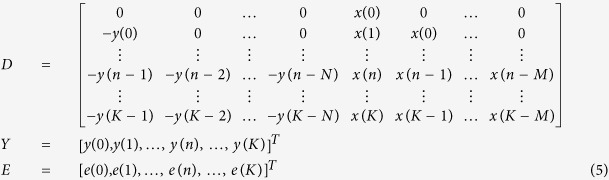


Thus, the solution for Θ is obtained by a linear regression that minimizes the norm of the error vector. The memory size N and M were chosen to maximize the goodness of fit in the test set. For the lateral and yaw speed we chose N = 7 (corresponding to a 35 ms memory), M = 7 (35 ms), the input *x* was the tail deflection. For the axial speed, because this kinematic parameter is mostly positive, we used the absolute value of the tail deflection, |*x*|, as the input with N = 20 (100 ms) and M = 7 (35 ms). The values obtained for the goodness of fit were 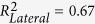, 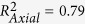 and 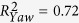.

We then tested the ability of the ARX model to fit unobserved trajectories. We split the dataset into two groups of equal size: large or small bouts, according to the maximal tail deflection in each bout. We assessed the goodness of fit of the ARX regression when generalizing between the small and large movements (see Table 1).

Table 1 shows that the axial speed trained on small tail bouts poorly predicted the kinematics of large tail bouts, probably because the measurement of axial displacement is noisier for small tail bouts than for large ones. In the other configurations, the goodness of fit of the regression trained on small movements and evaluated on large movements (and vice-versa) was close to the goodness of fit obtained when training on a mix of small and large bouts. These results demonstrate the ability of our method to predict the kinematics of trajectories outside the training dataset. The Python code required to extract the parameters Θ from a library of tail movements is available in the Jupyter Notebook (https://github.com/ajouary/VR_Zebrafish/blob/master/Code/PredictionTrajectory.ipynb) for reproducibility and reuse.

## Additional Information

**How to cite this article**: Jouary, A. *et al.* A 2D virtual reality system for visual goal-driven navigation in zebrafish larvae. *Sci. Rep.* **6**, 34015; doi: 10.1038/srep34015 (2016).

## Supplementary Material

Supplementary Information

Supplementary Movie 1

Supplementary Movie 2

Supplementary Movie 3

## Figures and Tables

**Table 1:** Each column contains the value of the goodness of fit for the three kinematic parameters evaluated using the ARX model.

	Trained on Small, Tested on Large	Trained on Large, Tested on Small	Trained on 80%, Tested on 20%
*R*^2^ of Lateral Speed	0.74	0.71	0.67 ± 0.05
*R*^2^ of Axial Speed	0.52	0.77	0.79 ± 0.1
*R*^2^ of Yaw Speed	0.72	0.71	0.72 ± 0.04

For the last column, the dataset was split randomly in a train and test dataset containing respectively 80% and 20% of the tail bouts. The goodness of fit was evaluated on the test dataset. This procedure was iterated 100 times leading to an estimate of the mean and the standard deviation for the goodness of fit.
