# Supplementary material for: A 2D virtual reality system for visual goal-driven navigation in zebrafish larvae
Source: Sci Rep. 2016 Sep 23;6:34015. doi: 10.1038/srep34015 (PMC5034285; doi:10.1038/srep34015)
Supplement: Supplementary Information [file srep34015-s1.pdf]

A 2D virtual reality system for visual goal-driven navigation in zebrafish larvae

Adrien Jouary[1], Mathieu Haudrechy[1], Raphaël Candelier[2], and German Sumbre[1,*]

[1]École Normale Supérieure, PSL Research University, CNRS, Inserm, Institut de Biologie de l'ENS (IBENS), F-75005 Paris, France
[2]Sorbonne Universités, UPMC Univ. Paris 06, UMR 8237, Laboratoire Jean Perrin, F-75005 Paris, France
[*]Institut de Biologie de l'ENS (IBENS), 46 rue d'Ulm, F-75005, France, sumbre@biologie.ens.fr

Movie Caption

1. The supplementary movie 1 illustrates the OMR experiments. The right panel shows the image of the larva superimposed on the grid projected below it. The left panel shows the orientation of the larva's heading direction relative to the movement of the grid. Two trials with different initial orientation are shown.
2. The supplementary movie 2 illustrates the prey-capture experiments when the feedback is provided in real-time. The right panel shows the video of the larva overlaid on a curved screen, the red region represents the virtual prey projected on the screen. The left panel shows the trajectory of the larva and its prey in the virtual environment. Two successful trials are shown.
3. The supplementary movie 3 illustrates the prey-capture experiments with a delayed feedback. Two unsuccessful trials are shown.

Supplementary Figure

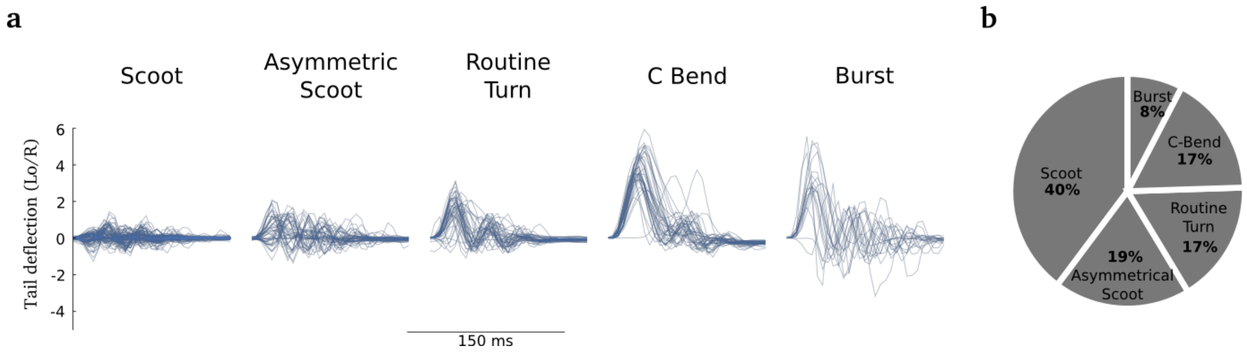

Figure 1. Supplementary Figure 1. Classification of tail movements. (a) Tail deflections for each category of movement from the library of tail bouts (N=306). Only the first 150 ms are shown. We displayed the opposite version of the tail deflection with negative values.**(b)** Proportion of the categories of movement in the library.

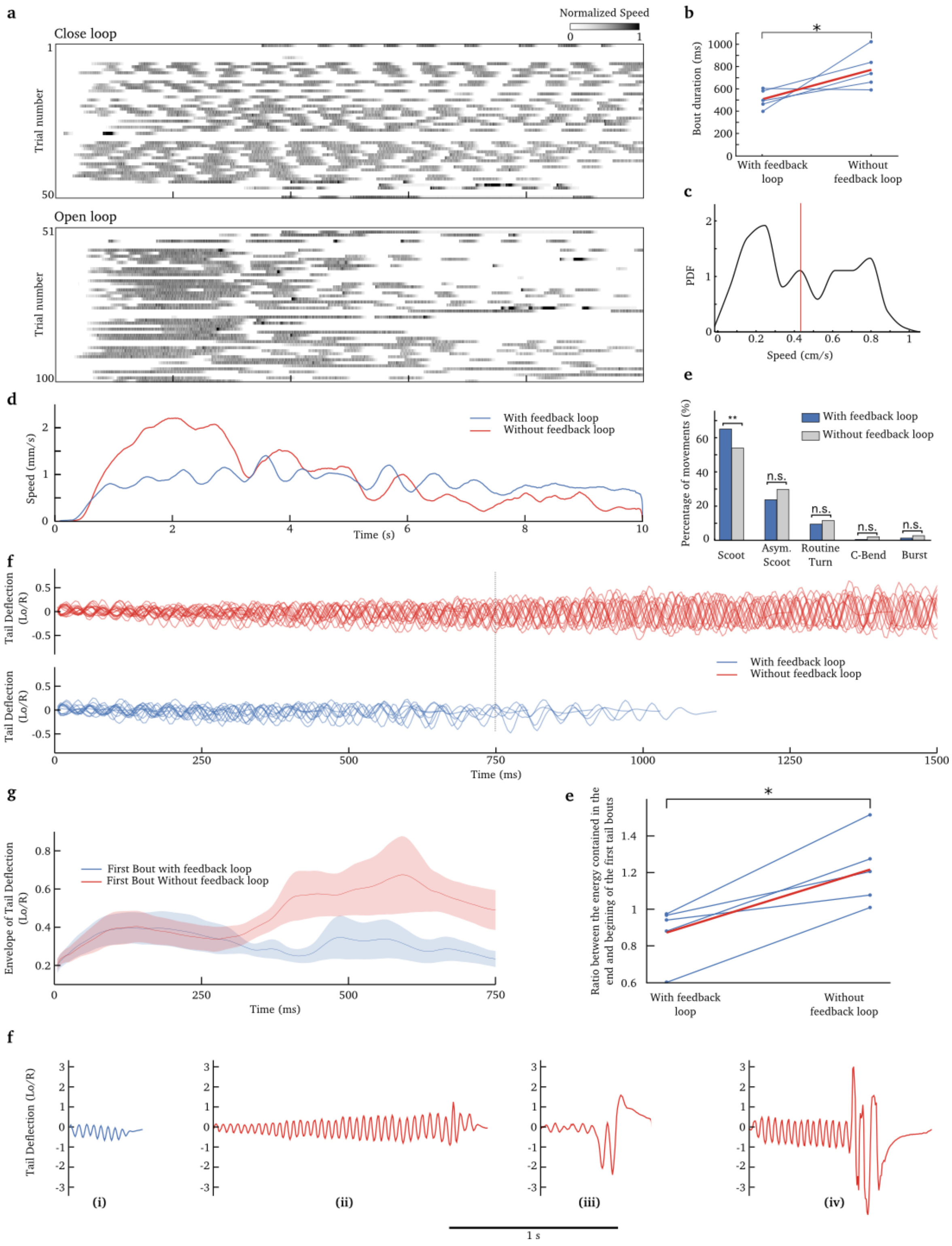

Figure 2. **Supplementary Figure 2. Modulation of the duration and amplitude of tail deflection during a bout**, legend next page

Figure 2. (Previous page.) **(a)** A representative example of the speed of the larva during an OMR experiment. Upper panel: 10 s trials in closed-loop conditions. Lower panel: 10 s trials in the absence of visual feedback. The open- and closed-loop trials were interleaved during the experiments. **(b)** Duration of bouts in the open- and closed-loop conditions (N=5). The average is shown in red. The duration of bouts was larger in open-loop conditions (770 ms) than closed-loop trials (517 ms, p=0,04, Wilcoxon signed-rank test). **(c)** Probability density of the average speed in the direction of the whole-field moving stimulus during trials, pooled across larvae. Only trials for which larvae swam for more than 500 ms were considered (N=148 trials from 5 larvae). The vertical red line shows the mean. **(d)** Average speed during the trials across larvae. In the closed-loop conditions, the larvae maintained a constant speed along the entire trial, while in the open-loop conditions, the larvae initially generated longer bouts, and their response to the stimulus thereafter decreased significantly. **(e)** Proportion of bouts for each category of movement in open- and closed-loop conditions (Not Significant: $p > 0.05$, $\star$: $p < 0.05$, $\star\star$: $p < 0.01$, $\star\star\star$:$p < 0,001$). **(f)** First tail bouts performed from 10 representatives trials, with feedback (blue) or in the absence of feedback (red). Note the increase in amplitude of the oscillation of the tail in the absence of feedback. **(g)** Average envelope of the oscillations of the tail deflection in the first bout of a trial for close-loop conditions (blue) or open-loop conditions (red). The shaded area corresponds to $\pm$ s.e.m. We show the first 750 ms (the maximal duration of bouts in close-loop conditions, dotted line in (e)). **(e)** Ratio of the signal energy between the end and the beginning of the fist tail bout of a trial ($\frac{\int_{D/2}^{D} T(t)^2\,\mathrm{d}t}{\int_0^{D/2} T(t)^2\,\mathrm{d}t}$, where T is the tail deflection and D the duration of the bout). Each line corresponds to one larva. The average is shown in red. In the presence of feedback, the ratio was close to 1, and significantly larger in its absence (p=0,03, Wilcoxon signed-rank test). For (f) and (g), we only considered forward scoot movements. **(f)** Examples of tail oscillations in single bouts. (i) a representative bout in the prescence of feedback (blue). (ii, iii, iv) examples of bouts showing on-line modulations of the tail deflection, in the absence of feedback. (ii) a progressive modulation of the tail deflection amplitude. (iii) a transition from a forward scoot to a routine turn at an early stage of the bout. (iv) a transition from a forward scoot to a burst at an later stage of the bout.
